# The Effect of Dietary Nitrate Supplementation on Isokinetic Torque in Adults: A Systematic Review and Meta-Analysis

**DOI:** 10.3390/nu12103022

**Published:** 2020-10-02

**Authors:** Ángel Lago-Rodríguez, Raúl Domínguez, Juan José Ramos-Álvarez, Francisco Miguel Tobal, Pablo Jodra, Rachel Tan, Stephen J. Bailey

**Affiliations:** 1 Faculty of Health Sciences, Universidad Isabel I, 09003 Burgos, Spain; angellagorodriguez@gmail.com; 2 Studies Research Group in Neuromuscular Responses (GEPREN), University of Lavras, 37200-000 Lavras, Brazil; 3 Faculty of Medicine, School of Medicine of Physical Education and Sport, Complutense University, 28040 Madrid, Spain; jjramosa@ucm.es (J.J.R.-Á.); miguelto@ucm.es (F.M.T.); 4 Faculty of Education Sciences, University of Alcalá, 19001 Guadalajara, Spain; pablo.jodraj@uah.es; 5 Faculty of Sports Medicine, Natural Sciences Division, Pepperdine University, Malibu, CA 90263, USA; rachel.tan@pepperdine.edu; 6 School of Sport, Exercise and Health Sciences, Loughborough University, Loughborough LE11 3TU, UK; s.bailey2@lboro.ac.uk

**Keywords:** beetroot, ergogenic aid, exercise performance, strength, muscle

## Abstract

Dietary nitrate (NO_3_^−^) supplementation, which can enhance performance in exercise settings involving repeated high-intensity efforts, has been linked to improved skeletal muscle contractile function. Although muscular strength is an important component of explosive movements and sport-specific skills, few studies have quantified indices of muscular strength following NO_3_^−^ supplementation, particularly isokinetic assessments at different angular velocities. We performed a systematic review and meta-analysis to determine whether dietary NO_3_^−^ supplementation improves peak torque, as assessed by the gold standard method of isokinetic dynamometry, and if this effect was linked to the angular velocity imposed during the assessment. Dialnet, Directory of Open Access Journals, MEDLINE, PubMed, SciELO, Scopus, and SPORTDiscus were searched for articles using the following search strategy: (nitrate OR beet*) AND (supplement* OR nutr* OR diet*) AND (isokinetic OR strength OR “resistance exercise” OR “resistance training” OR “muscular power”). The meta-analysis of data from 5 studies with 60 participants revealed an overall effect size of −0.01 for the effect of nitrate supplementation on isokinetic peak torque, whereas trivial effect sizes ranging from −0.11 to 0.16 were observed for independent velocity-specific (90°/s, 180°/s, 270°/s, and 360°/s) isokinetic peak torque. Four of the five studies indicated that dietary NO_3_^−^ supplementation is not likely to influence voluntary knee extensor isokinetic torque across a variety of angular velocities. These results suggest that NO_3_^−^ supplementation does not influence isokinetic peak torque, but further work is required to elucidate the potential of NO_3_^−^ supplementation to influence other indices of muscular strength, given the dearth of experimental evidence on this topic.

## 1. Introduction

Performance outcome in a variety of sports is determined by the ability to perform a range of rapid, dynamic, and explosive movements such as sprinting, changing direction, jumping, and exhibiting sport-specific skills (e.g., kicking, throwing, or hitting a ball). Indeed, the ability to produce force and power is considered among the most important factors for optimizing overall athletic performance [1,2]. It is important to note that there are a variety of methods to quantify force production (i.e., strength), such as isokinetic dynamometry, isometric assessments, resistance exercise protocols (e.g., one-repetition maximum (1RM), 3–10RM), and field tests (e.g., hand-grip and hand-held dynamometry, and body mass muscle testing such as vertical jump height) [3,4]. Each method provides different parameters of strength and has specific inherent weaknesses [5], such that strength assessments may not be equivalent between methods [4]. Although isokinetic strength measures have limited translation to sport-specific tasks, since these are rarely completed at an isokinetic pace [6], isokinetic assessments have several important advantages. These conducting assessments against a maximal resistance throughout the complete range of motion of a joint at different movement velocities [7], and the possibility of measuring torque [8]. In addition, isokinetic strength assessment is a highly reliable method for the measurement of force production [7,9,10]. Consequently, isokinetic dynamometry has been proposed as a gold standard method of strength assessment [11,12,13,14].

Dietary supplementation strategies (e.g., beta-alanine, sodium bicarbonate, and creatine) are often implemented in an attempt to enhance performance during single or intermittent maximal contractions; however, there are few supplements evidenced to be ergogenic in single intermittent maximal contractions [15]. One such supplement that may improve single maximal voluntary contractile function is inorganic nitrate (NO_3_^−^), which is typically administered as NO_3_^−^-rich beetroot juice (BR) [16]. Following ingestion, NO_3_^−^ is metabolized via the sequential reduction of NO_3_^−^ to nitrite (NO_2_^−^) and subsequently to nitric oxide (NO) [17], which plays a crucial role in skeletal muscle function [18]. The elevation in NO bioavailability following BR ingestion is thought to underpin the enhanced type II muscle fiber contractile function [18], lowered ATP cost of force production [5], and improved blood flow to the skeletal muscle [19,20]. More specifically, NO_3_^−^-induced enhancements to skeletal muscle contractile function may be due to improved calcium (Ca^2+^) handling [21,22].

The effect of NO_3_^−^ supplementation on involuntary electrically evoked contractions is equivocal. NO_3_^−^ supplementation has been reported to increase force production at low stimulation frequencies in some (≤20 Hz) [22,23,24], but not all studies [25,26]. Although dietary NO_3_^−^ supplementation has been reported to improve maximal voluntary contractile force during a mid-thigh pull [27], most studies have not observed an increase in maximal voluntary isometric contractile force when contracting the knee extensors [19,22,23,25,26,28,29]. Acute NO_3_^−^ ingestion has been reported to enhance peak torque during isokinetic dynamometry at high but not low angular velocities [30,31,32]. Type II muscle fibers are more likely to be recruited at high angular velocities [33,34] and NO_3_^−^ appears to be more effective at improving contractile function in type II muscle fibers [21]. This might account for the potential velocity-specific effects of NO_3_^−^ supplementation. Further evidence to support a potential velocity-specific effect of NO_3_^−^ supplementation is evidenced by increased peak power output [35,36,37] during a 30 s Wingate test performed using an inertial cycle ergometer, which results in faster pedaling cadence compared with an isokinetic cycle ergometer [38], and improved time to exhaustion when cycling at a high (115 rpm) but not a low (35 rpm) pedal cadence, after NO_3_^−^ supplementation [39]. However, there is currently a paucity of published reports examining the effect of NO_3_^−^ supplementation on torque at different angular velocities, and studies that have addressed this have yielded disparate findings [30,40]. Determining the potential of NO_3_^−^ supplementation to elicit velocity-specific effects on skeletal muscle contractile function is important to help inform exercise settings in which NO_3_^−^ supplementation is more and less likely to be ergogenic. Since isokinetic strength assessment is a reliable method for analyzing force production [10] at different movement velocities [7], it was selected as the experimental approach to assess the potential of NO_3_^−^ supplementation to improve skeletal muscle contractile function.

The aim of the present study was threefold: (i) to perform a systematic review of the studies that have investigated the effect of NO_3_^−^ supplementation on isokinetic torque production, (ii) to conduct a meta-analysis of reported findings, and (iii) to explore whether there is a velocity-specific effect of NO_3_^−^ supplementation on isokinetic torque production. It was hypothesized that NO_3_^−^ supplementation would improve isokinetic torque at high but not low angular velocities.

## 2. Methods

**Figure 1:**
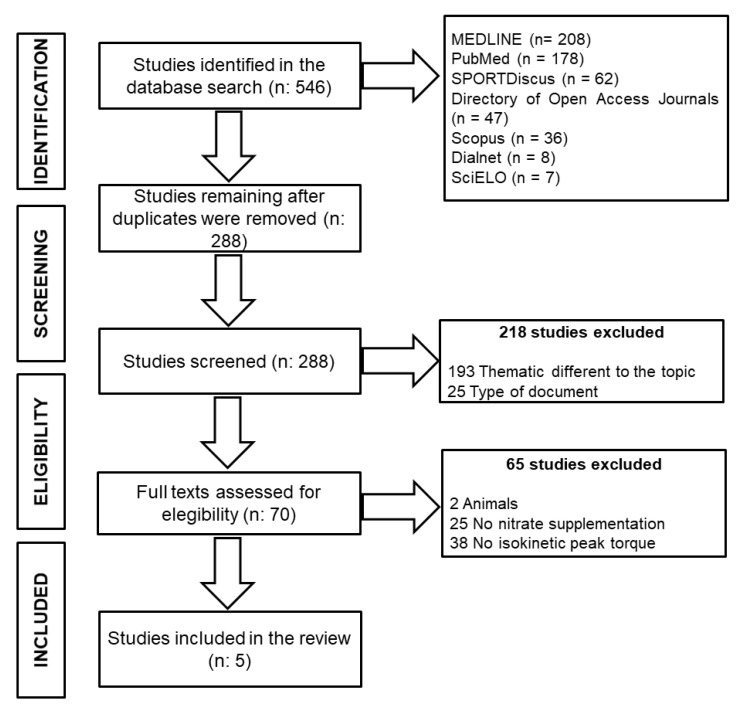
Preferred Reporting Items for Systematic Reviews and Meta-Analysis (PRISMA) flowchart.

The present systematic review and meta-analysis followed the Preferred Reporting Items for Systematic Reviews and Meta-Analysis (PRISMA) guidelines [41] and the PICOS (population, intervention, comparison, outcome, setting) criteria [42] and was conducted using Dialnet, Directory of Open Access Journals, MEDLINE, PubMed, SciELO, Scopus, and SPORTDiscus databases, including all results published before April 2020. The search strategy terms used were as follows: (nitrate OR beet*) AND (supplement* OR nutr* OR diet*) AND (isokinetic OR strength OR “resistance exercise” OR “resistance training” OR “muscular power”). To our knowledge, this is the first systematic review on this topic, and therefore the search did not include limitations of publication date or language. The original search yielded a total of 546 results. After the elimination of duplicates and screening of inclusion criteria, a total of 70 full-text articles were identified and reviewed by three authors (J.J.R.-Á., F.M.T., P.J.). A quality assessment was performed by two authors (J.J.R.-Á., F.M.T.) according to the PEDro scale [43]. A total of five articles met the eligibility criteria for the present systematic review and meta-analysis (Figure 1).

Based on the PICOS criteria, the following inclusion criteria were applied:

- Studies that were published as a full article and performed in adults (age 18–80 years);
- Studies that included a NO_3_^−^ and placebo intervention;
- Studies that assessed and reported isokinetic torque measures;
- Studies that employed a randomized double-blind experimental design.

Since this systematic review and meta-analysis was focused on isokinetic peak torque, we asked corresponding authors to provide maximum isokinetic torque data when they were not directly reported in the original article.

Meta-analytic statistical analysis was performed using Review Manager (RevMan) version 5.3 (Copenhagen: The Nordic Cochrane Centre, The Cochrane Collaboration, 2014). A fixed-effects model was applied and performed using mean and standard deviation of peak torque and the number of participants to quantify the standardized mean differences (SMDs) between NO_3_^−^ and placebo interventions, calculated as Hedges’ g [44]. The SMDs for each study were weighted as the reciprocal of their variance in order to calculate an overall effect and 95% confidence interval (CI), both for the overall movement velocities reported in the reviewed studies (i.e., overall analysis) and for each movement velocity reported at least in two studies (i.e., subgroup analysis). Effect sizes were defined as trivial (<0.2), small (<0.5), moderate (<0.8), and large (>0.8) [45]. *I*^2^ values were calculated for the percentage of total variation among studies [46]. *I*^2^ values were defined as small (25–50%), medium (50–75%), and large (>75%) [47].

The five studies included in the systematic review and meta-analysis comprised a total of 60 participants (38 males and 22 females, age 47 ± 18 years, BMI 26 ± 2 kg·m^−2^). All of the included studies provided acute administration of commercially available concentrated NO_3_^−^-rich beetroot juice (BR; Beet It Sport, James White Drinks, Ipswich, UK) 120 min prior to exercise (2 × 70 mL; ~11.2–13.4 mmol of NO_3_^−^) [30,31,32,48] or 180 min prior to exercise (1 × 70 mL; ~6.4 mmol of NO_3_^−^) [40]. 

## 3. Results

A summary of the methodologies and results of the studies included in this systematic review is provided in Table 1. Isokinetic dynamometry was used in all studies to assess voluntary peak torque production at various angular velocities ranging from stationary (0°/s) to fast movement (360°/s). In one study, knee extension and flexion were performed at 60°/s and 240°/s [40], respectively, while in the remaining studies, knee extensions were perfomed at 0°/s, 90°/s, 180°/s, 270°/s, and 360°/s [30,31,32,48]. 

There was an improvement in knee extension peak torque at a high angular velocity of 360°/s following NO_3_^−^ supplementation [31], but not during slower movement angular velocities of 60°/s [40], 90°/s [30,31,48], 180°/s [30,31,32,48], 240°/s [40], or 270°/s [30,31,32,48] (Table 1). There was no effect of NO_3_^−^ supplementation on knee flexion peak torque at slow- or fast-movement angular velocities of 60°/s or 240°/s respectively [40].

**Figure 2:**
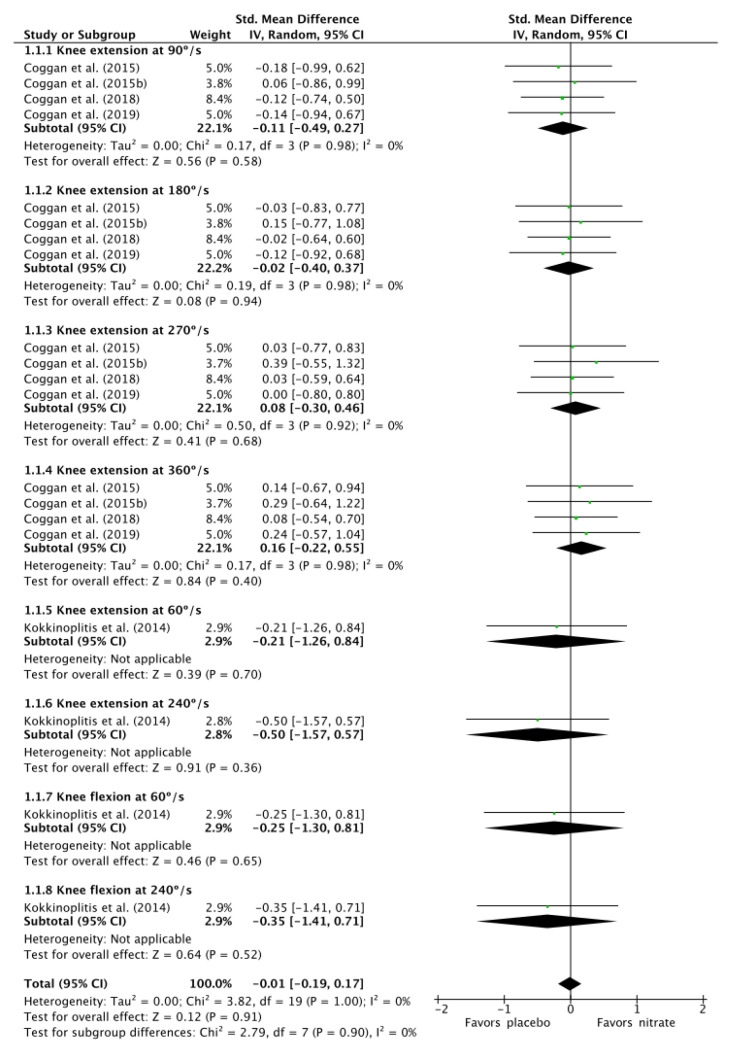
Forest plot comparing the effects of placebo and nitrate (NO_3_^−^) supplementation on isokinetic torque production. The size of the plotted squares reflects the statistical weight of the study. Horizontal lines denote the 95% confidence intervals.

There was a trivial effect size of −0.01 (CI: −0.19, 0.17; *I*^2^: 0%; *p* = 1.00; Figure 2) for the effect of NO_3_^−^ supplementation on isokinetic torque production when data from all angular velocities were combined. Subgroup analysis on the effect of NO_3_^−^ supplementation on knee extension isokinetic torque production at 60°/s, 90°/s, 180°/s, 240°/s, 270°/s, and 360°/s revealed that there was a trivial effect size of 0.01 (CI: −0.18, 0.19; *I*^2^: 0%; *p* = 1.00). Independent analysis for each angular velocity revealed trivial effect sizes (ESs) for the effect of NO_3_^−^ supplementation on knee extension isokinetic torque production at 90°/s (ES: −0.11, CI: −0.49, 0.27; *I*^2^: 0%; *p* = 0.98), 180°/s (ES: −0.02, CI: −0.40, 0.37; *I*^2^: 0%; *p* = 0.98), 270°/s (ES: 0.08, CI: −0.30, 0.46; *I*^2^: 0%; *p* = 0.92), and 360°/s (ES: 0.16, CI: −0.22, 0.5; *I*^2^: 0*%*; *p* = 0.98). 

## 4. Discussion

This is the first systematic review and meta-analysis to have investigated the potential influence of dietary NO_3_^−^ supplementation on isokinetic torque at different angular velocities. The main finding was that acute dietary NO_3_^−^ ingestion does not significantly alter isokinetic torque irrespective of the angular velocity imposed.

Of the five studies included in this review, only one study reported a significant ~11% improvement in knee extension peak torque at a high angular velocity of 360°/s [31], with two studies reporting a tendency for improved knee extension peak torque at 270°/s (+9%) [31] and 360°/s (+4%) [30] following acute NO_3_^−^ ingestion. In contrast, the remainder of the studies reported that knee flexion [40] and knee extension peak torque at slow-to-moderate angular velocities were not influenced by NO_3_^−^ supplementation [30,31,32,40,48]. Therefore, the findings from the present meta-analysis suggests that acute dietary NO_3_^−^ ingestion does not influence lower limb muscle isokinetic torque.

It is recognized that the increase in plasma [NO_2_^−^] following NO_3_^−^ supplementation is a correlate of improved performance [48,49]. Since plasma [NO_2_^−^] attains peak values ~3 h post NO_3_^−^ ingestion [49,50,51], the lack of an effect of NO_3_^−^ ingestion on peak torque across the range of angular velocities assessed in the current meta-analysis might be a result of the acute NO_3_^−^ ingestion occurring 2 h prior to the peak torque assessments [30,31,32,40,48] such that a suboptimal plasma [NO_2_^−^] was attained. It should also be highlighted that a limitation of some of the studies included in the current meta-analysis is that plasma [NO_2_^−^] was either not measured [30,40], or was measured with an assay with insufficient sensitivity to detect an increase in plasma [NO_2_^−^] post NO_3_^−^ ingestion [31]. In addition, since all studies in the current meta-analysis assessed the effect of acute NO_3_^−^ ingestion on peak torque at different angular velocities, and since there is evidence to suggest that chronic NO_3_^−^ supplementation may be more ergogenic than acute NO_3_^−^ ingestion (at least during endurance exercise [52,53]), chronic NO_3_^−^ supplementation may have elicited enhanced isokinetic peak torque production. It is possible that chronic NO_3_^−^ supplementation can elevate skeletal muscle tissue stores of NO_3_^−^ and NO_2_^−^ compared to acute NO_3_^−^ ingestion [54], which could facilitate improved skeletal muscle contractile performance. Moreover, chronic NO_3_^−^ supplementation has been reported to increase the content of the calcium-handling proteins calsecuestrin (CASQ) and the dihydropyridine receptor (DHPR) in skeletal muscle, and evoked contractile force in mouse fast-twitch muscle, which supports the postulate that chronic NO_3_^−^ supplementation could improve skeletal muscle contractile function [21]. Although an increase evoked for production was reported after chronic NO_3_^−^ supplementation in humans, this was not accompanied by increases in skeletal muscle CASQ and DHPR content [22]. In addition, the lack of an effect as reported in the study by Kokkinoplitis and Chester [40] compared to the studies by Coggan and colleagues [30,31,32,48] could be a function of the lower NO_3_^−^ dose (6.4 mmol) administered in the former study. Indeed, it was reported that exercise performance dose-dependently increases after acute NO_3_^−^ supplementation up to 8.4 mmol, with no additional performance benefits at 16.8 mmol compared to 8.4 mmol, at least during endurance exercise [49]. Further research is required to address the effects of chronic NO_3_^−^ supplementation on skeletal muscle contractile function and the potential mechanisms that could underpin an ergogenic effect. 

It is becoming increasingly appreciated that the efficacy of NO_3_^−^ supplementation is influenced by the population evaluated, which could be related to factors influencing NO bioavailability [18,55]. For example, basal NO synthesis is higher in individuals with a high cardiorespiratory fitness compared to that in senescent populations [56] and lower in individuals with a pathology [57,58]. There is also evidence that the increase in plasma [NO_2_^−^] (which is a sensitive NO biomarker [59]) after NO_3_^−^ supplementation is correlated to relative improvements to maximal knee extensor power (*R* = 0.60) [48]. Peak torque was enhanced to a greater extent in heart failure patients [31] compared to young and older healthy individuals [30,32,40,48] after NO_3_^−^ supplementation. This might be a function of impaired NO synthesis in heart failure patients, providing a greater scope for NO_3_^−^ supplementation to increase NO synthesis and muscle contractile performance compared to healthy individuals with a higher residual NO synthesis and muscle function. Furthermore, sex differences may influence the efficacy of NO_3_^−^ supplementation. Indeed, Coggan et al. [48] observed that females tended to have a greater magnitude of increase in maximal power after NO_3_^−^ supplementation, which might be linked to the greater increase in plasma [NO_2_^−^] after NO_3_^−^ supplementation in females [50]. Since the efficacy of NO_3_^−^ supplementation appears to be linked to NO synthesis, and since various subject population characteristics including health, age, and sex can modulate NO bioavailability, further research is required to aid understanding of the different settings in which NO_3_^−^ supplementation might influence muscular strength.

It should be noted that there are a variety of methods to assess muscle contractile function; these methods range from isometric to the commonly implemented isotonic methods. In addition, there are methods that employ single-joint (isokinetic dynamometry) or multi-joint movements (1RM testing), which may have better reliability, sensitivity, and validity, while other methods may better translate to sport-specific performance [4]. Given the relative strengths and weaknesses of each approach, future studies should seek to implement multiple modes of assessment for strength in order to more clearly resolve the potential influence of NO_3_^−^ supplementation on the muscle contractile function. It should also be acknowledged that although NO_3_^−^ supplementation does not appear to influence isokinetic peak torque during independent angular velocity assessments, subsequent calculations of maximal power and velocity of contraction from peak torque values obtained across a series of independent angular velocity assessments were improved by NO_3_^−^ supplementation [30,31,32,48]. In addition to isokinetic torque, the effect of NO_3_^−^ supplementation on different aspects of skeletal muscle contractile function is equivocal. Indeed, NO_3_^−^ supplementation has been reported to increase force production at low stimulation frequencies in some (≤20 Hz) [22,23,24] but not all studies [25,26]. Although most studies have not observed an increase in maximal voluntary isometric contractile force after dietary NO_3_^−^ supplementation [19,22,23,25,26,28,29], improved weightlifting performance [30,31,32,48,60] and concentric and eccentric contractile force during the back squat [61] have been reported after dietary NO_3_^−^ supplementation. There is also evidence of an increased peak power output during a 30 s Wingate test after dietary NO_3_^−^ supplementation [35,36,37]. Therefore, the possibility of NO_3_^−^ supplementation improving skeletal muscle strength, power, and velocity cannot be excluded on the basis of the present meta-analysis, and further studies are warranted to provide greater clarity on the effects of NO_3_^−^ supplementation on skeletal muscle contractile function.

## 5. Conclusions

In conclusion, the current systematic review and meta-analysis indicates that acute dietary NO_3_^−^ ingestion is not likely to induce positive benefits to muscle peak torque production at a variety of angular velocities in the lower limbs, at least when assessed using isokinetic dynamometry. The lack of an effect of NO_3_^−^ supplementation might be linked to NO bioavailability, which is modulated by factors such as dosing strategy, and participant health and training status, and sex. Given the paucity of literature, further research is required for a more complete understanding of the influence of NO_3_^−^ supplementation on different aspects of muscle strength.

## Figures and Tables

**Table 1:** Studies assessing the effects of dietary NO_3_^−^ supplementation on isokinetic peak torque production in humans.

Reference	Subjects	Supplementation	Exercise Protocol	Findings (BR vs. PL)
Coggan et al. [30]	7 M/5 F healthy adults	120 min prior to exercise ingestion of 2 × 70 mL BR shots (~11.2 mmol NO_3_^−^)	Isokinetic knee extension peak torque at: 90°/s, 180°/s, 270°/s, and 360°/s	−1.86% at 90°/s −1.72% at 180°/s +0% at 270°/s +4.27% at 360°/s
Coggan et al. [31]	5 M/4 F heart failure patients	120 min prior to exercise ingestion of 2 × 70 mL BR shots (~11.2 mmol NO_3_^−^)	Isokinetic knee extension peak torque at: 90°/s, 180°/s, 270°/s, and 360°/s	+1.47% at 90°/s −3.81% at 180°/s +9.41% at 270°/s +10.94% at 360°/s *
Coggan et al. [48]	13 M/ 7 F healthy young and older adults	120 min prior to exercise ingestion of 2 × 70 mL BR shots (~12.3 mmol NO_3_^−^)	Isokinetic knee extension peak torque at: 90°/s, 180°/s, 270°/s, and 360°/s	−3.11% at 90°/s −0.67% at 180°/s +0.8% at 270°/s +3.06% at 360°/s
Coggan et al. [32]	6 M/6 F healthy older adults	120 min prior to exercise ingestion of 2 × 70 mL BR shots (~13.4 mmol NO_3_^−^)	Isokinetic knee extension peak torque relative to body mass at: 0°/s, 90°/s, 180°/s, 270°/s, and 360°/s	−2.06 at 0°/s −2.82% at 90°/s −2.94% at 180°/s +0% at 270°/s +8.33% at 360°/s
Kokkinoplitis and Chester [40]	7 M healthy adults	180 min prior to exercise ingestion of 1 × 70 mL BR shot (~6.4 mmol NO_3_^−^)	Isokinetic knee extension and flexion peak torque at: 60°/s and 240°/s	Knee extension −3.47% at 60°/s −5.56% at 240°/s Knee flexion −6.85% at 60°/s −13.83% at 240°/s

* = significant difference between BR and PL; BR: beetroot juice; PL: placebo juice; F: females; M: males; min: minutes; NO_3_^−^: nitrate; s: seconds; °: degrees.
